# Comparing specialist medical care with specialist medical care plus the Lightning Process® for chronic fatigue syndrome or myalgic encephalomyelitis (CFS/ME): study protocol for a randomised controlled trial (SMILE Trial)

**DOI:** 10.1186/1745-6215-14-444

**Published:** 2013-12-26

**Authors:** Esther Crawley, Nicola Mills, Will Hollingworth, Zuzana Deans, Jonathan A Sterne, Jenny L Donovan, Lucy Beasant, Alan Montgomery

**Affiliations:** 1 Centre for Child and Adolescent Health, School of Social and Community Medicine, Oakfield House, Oakfield Grove, Bristol, BS8 2BN, UK; 2 School of Social and Community Medicine, Canynge Hall, Whatley Road, Bristol BS8 2PS, UK; 3 School of Social and Community Medicine, Oakfield House, Oakfield Grove, Bristol BS8 2BN, UK; 4 University of Nottingham Clinical Trials Unit Queen’s Medical Centre, Nottingham NG7 2UH, UK

**Keywords:** Fatigue, Paediatrics, Chronic fatigue syndrome, Myalgic encephalomyelitis

## Abstract

**Background:**

Chronic fatigue syndrome or myalgic encephalomyelitis (CFS/ME) is a relatively common and potentially serious condition with a limited evidence base for treatment. Specialist treatment for paediatric CFS/ME uses interventions recommended by National Institute for Health and Clinical Excellence (NICE) including cognitive behavioural therapy, graded exercise therapy and activity management. The Lightning Process® (LP) is a trademarked intervention derived from osteopathy, life-coaching and neuro-linguistic programming, delivered over three consecutive days as group sessions. Although over 250 children with CFS/ME attend LP courses each year, there are no reported studies on the effectiveness or cost-effectiveness.

**Methods:**

This pragmatic randomised controlled trial is set within a specialist paediatric CFS/ME service in the south west of England. Children and young people with CFS/ME (n = 80 to 112), aged 12 to 18 years old will be randomised to specialist medical care (SMC) or SMC plus the LP. The primary outcome will be physical function (SF-36 physical function short form) and fatigue (Chalder Fatigue Scale).

**Discussion:**

This study will tell us whether adding the LP to SMC is effective and cost-effective compared to SMC alone. This study will also provide detailed information on the implementation of the LP and SMC.

**Trial registration:**

Current Controlled Trials ISRCTN81456207 (31 July 2012).

## Background

The Royal College of Paediatrics and Child Health in the UK has defined chronic fatigue syndrome or myalgic encephalomyelitis (CFS/ME) as ‘generalised fatigue, causing disruption of daily life, persisting after routine tests and investigations have failed to identify an obvious underlying “cause”’ [1]. The NICE guidelines recommend a minimum time of three months of fatigue before making a diagnosis in children [2].

CFS/ME in children is a relatively common [3-6] and potentially serious condition with over 50% of children bedbound at some stage and a mean time off school of one academic year [7].

There is a limited evidence base for the treatment for children with CFS/ME. There is one randomised controlled trial (RCT) comparing cognitive behavioral therapy (CBT) (n = 29) and waiting list (delayed CBT) (n = 33) which reported improvements in fatigue, physical function and return to full time school in the early CBT arm [8,9]. However, in this study, approximately 40% of those in the early CBT arm did not have an improvement at five months. In an RCT comparing family-focussed CBT versus psycho-education [10] (n = 63) children in the CBT group were attending more school at discharge and three months post treatment but not at the primary outcome time point (6 months) [10]. Long-term follow-up (24 months) in this group showed that recovery (defined using fatigue and school attendance) was 79% in the family-focused CBT group compared with 64% in the psycho-education group [11]. A recent RCT comparing Internet based CBT (n = 68) with usual care (n = 67) showed more children in the CBT arm (75% versus 16%) attended full time school, did not have severe fatigue and had normal physical functioning (78% versus 20%) at six month follow-up [12].

The Phil Parker Lightning Process® (LP) is a trademarked intervention that is used for a variety of conditions including CFS/ME. It has been developed from osteopathy, life coaching and neuro-linguistic programming. The intervention includes three group sessions on consecutive days where young people are taught skills that they can try out for themselves including looking at their sitting and standing posture. Families currently pay approximately £620 to attend the LP course.

Even though over 250 children and young people a year use the LP as an intervention for their CFS/ME, there are currently no reported studies investigating the effectiveness or possible side effects (for example serious adverse events) of the LP.

This trial continues from the SMILE feasibility study which showed that recruitment, randomisation and data collection on health resource use was feasible and acceptable [13]. In this trial we will compare the effectiveness and cost-effectiveness of specialist medical care (SMC) plus the LP with SMC alone for children with CFS/ME.

### Hypothesis

We will test the null hypothesis that the addition of the LP to SMC has no additional benefit to SMC alone and is not cost-effective.

## Method

### Design

**Figure 1:**
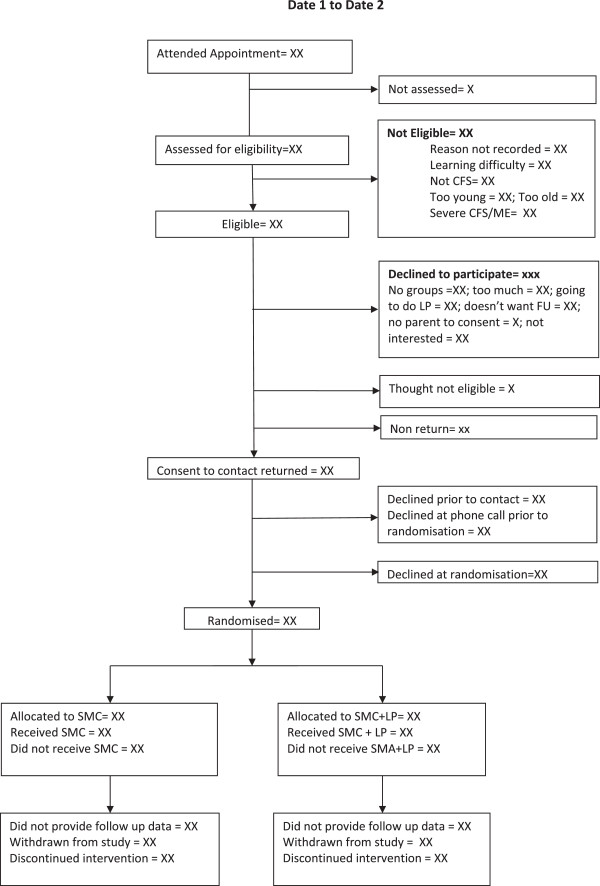
SMILE flow diagram.

This is a pragmatic [14] randomised controlled trial comparing SMC plus the LP with SMC alone among children with CFS/ME. Qualitative research methods have been integrated into this study to ensure clear understanding of the processes (implementation, acceptability and setting of the interventions). See Figure 1 for trial flow diagram.

### Population

Children and young people aged 12 to 18 years inclusive will be recruited after assessment by the Bath/Bristol paediatric CFS/ME service. This is a large regional and national service that currently provides assessment and treatment for over 250 children a year. The majority of referrals are from South Gloucestershire, Bristol, Somerset and West Wiltshire. Referrals are made by paediatricians, general practitioners and, in some cases, schools. Children are given a diagnosis of CFS/ME after a thorough assessment and screening blood tests according to guidelines produced by the National Institute for Health and Care Excellence (NICE) [2]. The majority of children referred into the service have CFS/ME as other causes of fatigue are usually excluded prior to referral. Approximately 10% of children referred into the service are housebound and are assessed at home.

### Inclusion/exclusion criteria

Children will be included if they have CFS/ME and are between 12 and 18 years old inclusive. Children will be excluded if: they are too severely affected to attend hospital appointments (defined as children and young people that do not regularly leave their house); or if they or their parents have insufficient English to either understand the patient information sheet and consent form to take part in the LP or the research interviews.

### Recruitment

Potentially eligible children and their families will be identified by the clinician conducting the initial clinical assessment who will inform them about the study and give both the young person and their parents patient information sheets. The clinician will obtain consent from the young person and their family to be contacted by a researcher. If willing, the recruiting researcher will contact the family and arrange to visit them at a convenient location (usually at home) to discuss and provide further information about the study.

### Randomisation

The recruiting researcher will explain the rationale for the study and its design, the uncertainties about the effectiveness of either intervention, the known advantages/disadvantages of the interventions, the options available outside the RCT, and the right not to take part in the study or to withdraw at any time. Willingness to participate will be ascertained, checking that both the young person and their family understand the study. Those willing to take part in the study will be asked to consent to randomisation and sign the consent form. The recruiting researcher will then telephone the automated randomisation service operated by the Bristol Randomised Trials Collaboration for the intervention allocation, which will be conveyed to the participant. Allocation will be minimised [15] by age and gender, and retain a random component to reduce risk of prediction of allocation. If for any reason the service is unobtainable, randomisation will be completed during the next working day and the participant will be told of the results by phone or in person.

### Interventions

•SMC: children and their families are offered a variety of treatment options that are recommended in NICE guidelines [2]. Typically this is centred around graded activity and involves a follow-up phone call at two weeks followed by family based rehabilitation consultations lasting one hour at approximately six weeks, three months, and four and a half months. The number and timing of the sessions are agreed with the child and family and varies depending on the needs and goals of the child. Children who have high levels of anxiety are offered three individual sessions of CBT every two weeks over a six week period. Other interventions such as graded exercise therapy (GET) [16] are available for children and young people if needed. The clinical team providing SMC are not informed by the research team to which trial arm a participant has been allocated.

•SMC plus the LP: in addition to the SMC detailed above, young people and their parents will be asked to read the information about the LP on the website [http://www.lightningprocess.com] or using information sheets. If the young person is well enough, they will be asked to read a book about the LP, given to them from the LP team, or listen to an audio book if preferable. Children/young people and their parents will be asked to complete an assessment form (which will take about ten minutes) where they are asked to identify their goals and describe what they learnt from reading the book. After this they will have a telephone call with a LP practitioner (usually approximately 20 minutes). This is used to check that the young person and their parents are happy about attending the course, checks the goals identified by the young person and is an opportunity for the young person and their parents to ask further questions. If the young person and their family are happy to continue, the young person will be given a date to attend a course.

•The course is three sessions on three consecutive days. Each session is three hours and forty-five minutes long. Group sessions will include three to five young people between 12 and 18 years of age who live within the region covered by the CFS/ME service. During the group, children and young people will receive a theory session and a practical session. The course is free to those participating in the trial.

1. The theory session will include taught elements on the stress response, how the mind-body interacts and how thought processes can be both helpful and negative. The language used by young people will be discussed and in some cases challenged. Young people will be encouraged to think about what they may be able to take responsibility for and change. The taught sessions are followed by a group discussion.

2. The practical session is used to put some of the skills learnt into practice. Young people identify a goal they wish to achieve (such as standing for longer) and are then given alternative ways to think about and prepare for this. This involves using different cognitive (thinking) strategies before and during the period in which achieving the goal is attempted. Young people are also asked to identify a goal wherein they can practise the strategies in the afternoon or evening. This goal will usually be short but could be an activity that is up to 30 minutes long.

3. The LP practitioner will then arrange two follow-up phone calls with the young person and parents within two weeks of the course and then approximately six to eight weeks later.

### ‘Outcome assessment’

The following self-completed inventories will be collected at the first clinical assessment (baseline), 3, 6 and 12 months: 11 item Chalder Fatigue Scale [17]; pain visual analogue scale; physical function short form (SF-36) [18]; the Spence Children’s Anxiety Scale (SCAS) [19]; the Hospital Anxiety and Depression Scale (HADS) [20] for children aged 14 and over, and the Euroqol (EQ-5D) [21] a five-item quality of life inventory. Children and young people are asked about school attendance and home tuition in a two-item inventory. We will ask for consent to check school attendance using school records at assessment, 3, 6 and 12 months. Follow-up questionnaires will be sent in the post with a stamped addressed envelope for self completion.

#### Primary outcome

The primary outcome will be the SF-36 physical function subscale analysed as a continuous variable collected at six months post-randomisation. The SF-36 will be scored as the sum of responses to the ten items, each of which is coded 0 for ‘Yes, limited a lot’, 5 for ‘Yes, limited a little’ and 10 for ‘No, not limited at all’. This yields a score varying from 0 (for the highest level of disability) to 100 for no disability. The SF-36 physical function subscale will be used as we are primarily interested in change in physical function. It has been used in studies of adolescent CFS/ME [22] and in CFS trials in adults [16]. We chose six months as this was the first time that all those randomised to LP and SMC will have received LP. Secondary outcome measures will be school attendance, calculated as a percentage of expected sessions, at 3, 6 and 12 months; the SF-36 (physical function) at 3 and 12 months; the Chalder Fatigue Scale score at 3, 6 and 12 months; pain visual analogue scale at 6 months and quality adjusted life years (QALYs, derived from the EQ-5D) over the 12 month follow- up period.

### Measures used for economic evaluation

Parent(s)/guardian(s) will be asked to complete three inventories at the start of the trial (just after randomisation) and at 3, 6 and 12 months. These include an adapted four-item Work Productivity and Activity Impairment Questionnaire: General Health V2.0 (WPAI:GH) [23] and a resource use questionnaire assessing health service (for example, GP or specialist care), educational service (for example, school counsellor) use and travel costs most relevant to the CFS/ME population (all included in Additional file 1 and Additional file 2).

#### Non-responders

Those who have not replied within one week of each mail out will be sent a reminder letter requesting that the original set of questionnaires is completed and returned. A reduced set of questionnaires (comprising SF-36, Chalder Fatigue Scale score and school attendance inventory) with a stamped addressed envelope will also be included in case the originals have been mislaid. After another two weeks, those not returning any questionnaires will be telephoned by a researcher who will invite the respondent to complete the reduced questionnaire set over the telephone.

### Statistical considerations

#### Sample size

We used a definition of a clinically important difference for the SF-36 physical function subscale from three expert consensus panels for chronic diseases in adults. The panels conducted a literature search and used the Delphi technique to reach consensus on the thresholds for change over time for small, moderate and large clinically important SF-36 change scores [24]. Consensus was agreed by each panel that a small clinically important difference would be 10 as this is the equivalent to two state changes (a state change is one improvement in one item - the minimum difference between inventories). A moderate improvement was defined as 20 and a large improvement as 30.

To be able to detect a difference between the two treatment arms of 8 to 10 points on the SF-36 PCS at six months with 90% power and 1% two-sided alpha, we have calculated (using STATA) that a total of 32 to 50 participants in each arm for analysis are required. Allowing for 10 to 20% non collection of primary outcome data at six months, we aim to recruit 80 to 112 participants to the study.

#### Data analysis

The analysis and presentation of the trial will be in accordance with CONSORT guidelines [14]. A full statistical analysis plan will be developed and agreed with the Trial Management Group and Trial Steering Committee prior to conducting any analyses.

We will use descriptive statistics to compare characteristics of invited individuals who did or did not agree to take part and eligible individuals who were randomised or not randomised. We will also examine the balance between the trial arms in participant characteristics.

The primary intention-to-treat analysis will compare the SF-36 physical function subscale at six months between groups using multivariable linear regression adjusting for baseline value of the outcome and minimisation variables, paying attention to 95% confidence intervals as well as *P*-values. Similar analyses using appropriate regression models will be conducted for secondary outcomes. Sensitivity analyses will be conducted using standard techniques to impute missing data [25], and to estimate the efficacy of the intervention among compliers.

The statistical analysis plan including subgroup analyses for the primary outcome. This will be conducted to explore differences in outcome according to age (< 14.99 versus 15.0 to 17.99), gender (male/female) and severity (no school attendance versus some school attendance) as age and gender are risk factors for CFS/ME and disease severity is a predictor of outcome. These subgroup analyses are exploratory and will be interpreted with due caution [26]. Any suggestion that the intervention effect differs according to age, gender or severity would need to be confirmed in subsequent studies. These will include interaction terms in regression models.

### Economic evaluation

The economic evaluation will gather information on the costs to the NHS, other government agencies and wider society of the interventions in the two arms of the RCT. The primary cost-effectiveness analysis will compare incremental differences in NHS (including LP referral costs) and other public sector costs with any health gains achieved over 12 months, measured in QALYs. We will estimate the net monetary benefit of specialist medical care plus the LP compared to specialist medical care alone and quantify uncertainty using confidence intervals, cost-effectiveness acceptability curves and sensitivity analyses [27]. In secondary analyses we will tabulate the wider costs, including travel and productivity costs and consequences, including school time missed, in a cost consequence study [28,29].

### Qualitative research

A process evaluation, drawing on qualitative research methods, will be undertaken to assess the implementation, acceptability and setting of the intervention as well as to explore users’ and practitioners’ views and experiences of both the intervention and outcomes.

#### In-depth interviews

Interviews with parents and children/young people were conducted at three time points for the feasibility study: after initial assessment but prior to randomisation, after randomisation but prior to the intervention and after the intervention. Analyses of data will continue from the feasibility study, supplemented with further in-depth interviews undertaken with some parents and children after the intervention to further assess their experiences of the trial and intervention. Interviews will be semi-structured in that they will follow a checklist of topics to ensure consistency, but parents and children will be able to raise issues of importance. Interviews will explore the recruitment process, including views and experiences of the initial assessment and recruitment to trial appointments, the written and verbal information provided to potential participants and its acceptability, and reasons for accepting or declining participation; beliefs, expectations and preferences about interventions in the early stages of the trial, and experiences of interventions and outcome later on; and prior exposure and external influences to the intervention that might impact upon its implementation and effectiveness.

#### Recording of recruitment to trial consultations

All recruitment consultations will be audio-recorded to document the interaction between recruiter and potential participant to explore information provision, recruitment techniques, patient treatment preferences, and randomisation decisions to identify recruitment difficulties and support change. This novel method can provide essential information about the way the study and its interventions are perceived as well as optimising methods for recruitment and design. It has proved crucial in evaluating information exchange and improving informed consent and rates of randomisation/acceptance of allocation in previous studies [30-32].

#### Observations

We will analyse observation data already collected on a small number of interventions, specialist medical assessments and specialist medical treatment follow-up sessions observed by the qualitative researcher to assess the implementation, acceptability and setting of intervention/treatment provision. Detailed notes have been taken, including the context, intensity and variability of intervention/treatment delivery, to understand how intervention/treatment is delivered and received in practice and to help interpret outcome results (for example, variations of effects in subgroups). All intervention sessions were audio-recorded, with consent, for monitoring purposes. We may observe further sessions if necessary following analysis of existing data. The final number of observations will be determined by data saturation, although up to ten observations in total are anticipated.

#### Qualitative data analysis

Analysis will be an ongoing and iterative process commencing soon after data collection. It will build on data analysed from the feasibility study and will inform further sampling and data collection. Interview transcripts and observation notes will be imported into NVivo (QSR International) [http://www.qsrinternational.com/products_nvivo.aspx: 2011] where they will be systematically assigned codes and analysed thematically to identify themes using techniques of constant comparison. Individuals exhibiting contrasting attitudes (‘negative cases’) will be studied in detail to understand reasons underlying such contrasts and to gain a deeper understanding of the data and findings. Throughout analysis, the perspectives of the individuals will be paramount, with careful account taken of the context within which the discussion takes place. Descriptive accounts will be produced, and theoretical explanations for behaviours, opinions and decisions will be developed.

Content analytic methods will be used to describe in a structured manner what was said by whom and how often in the consent to randomisation appointments. More flexible grounded theory methods (as in the interviews above) will be applied to identify common or divergent themes, particularly focussing on the impact of statements by the recruiter on patients. Conversation analysis will be used to focus in great detail on certain sections of the transcripts, for example the interactions during which randomisation is offered.

### Data protection

Children and young people are allocated a unique 13 digit identification number made up of the centre number, the team number, an individual patient number, first four digits of the postcode, and patient initials. This number is assigned to the patient and is used on assessment forms prior to transfer of data so they are anonymised at source. A list of names and corresponding identification numbers are kept separately and securely on a password protected NHS server.

Audio-recordings will be encrypted, password protected and stored on a secure university server for five years. This is to enable us to check recordings if necessary while reports are being written. Transcripts will be anonymised and secure password protected university server.

### Data monitoring

The data monitoring group will receive notice of serious adverse events (SAEs) for the sample as whole. If the incidence of SAEs of a similar type is greater than would be expected in this population, it will be possible for the data monitoring group to receive data according to trial arm to determine any evidence of excess in either arm.

Primary outcome data at six months will be examined once data are available from 50 patients, to ensure that neither arm is having a detrimental effect on the majority of patients. An independent statistician with no other involvement in the study will investigate whether more than 20 participants in the study sample as a whole have experienced a reduction of ≥ 30 points on the SF-36 at six months. In this case, the data will then be summarised separately by trial arm, and sent to the data monitoring group for review. This process will ensure that the trial team will not have access to the outcome data separated by treatment arm.

### Ethical review

A favourable ethical opinion was given on 8 September 2010 (reference 10/H0206/32) by South West 2 Local Research Ethics Committee. Two favourable opinions have been provided on 31 May 2011 and 6 September 2012 for amendments to study documents and protocol.

All children, young people and parents/carers involved in the study provided consent or assent (under 16 years). Parents of children providing assent also provided consent to take part in the study.

## Discussion

Paediatric CFS/ME is a relatively common and frequently disabling condition with limited treatment options of proven efficacy available within the NHS. Although the LP is popular, it currently has anecdotal evidence to support its efficacy as no formal studies have as yet been carried out. It is important for parents, children with CFS/ME and the NHS to know whether the LP is more effective and cost-effective when used with NHS SMC rather than existing NHS SMC alone.

CFS/ME is different in children and adults with different risk factors [33-35], course and outcome [36]. It is therefore not possible to complete a study in adults and extrapolate the results to children. Children and families will also be followed-up closely during and after the LP intervention. The interviews with parents at different stages of the study will help us understand parental and young people’s views at each stage of the process.

Because the participants will be young people, we have put in place rigorous procedures for informed consent from young people and their parent(s)/guardian(s) if they are < 16 years old. In the clinic, the clinician will ask for consent/assent for contact by a researcher and qualitative researcher. Consent/assent to the study and to randomisation will be obtained by a researcher after a full explanation of the study when both the young person and the family have had sufficient opportunity to ask questions. Young people and their families will be given as long as they need before giving consent/assent within the confines of the study. We will then obtain further consent/assent prior to each interview to check that young people or their parents continue to be willing to participate. We will also obtain consent/assent prior to recording any interventions from all present.

Qualitative data collected to date demonstrate that both children and their families find the recruitment process acceptable, the information given of high quality and both children and their parents have been grateful they were part of the study.

### Trial status

On going trial.

## Abbreviations

CFS: Chronic fatigue syndrome; CBT: Cognitive behavioral therapy; EQ-5D: Euroqol Questionnaire; GET: Graded exercise therapy; HADS: Hospital Anxiety and Depression Scale; LP: Lightning Process®; ME: Myalgic encephalopathy; NHS: National Health Service; NICE: National Institute for Health and Clinical Excellence; QALY: Quality Adjusted Life Year; RCT: Randomised controlled trial; SAE: Serious adverse events; SCAS: Spence Childrens Anxiety Scale; SD: Standard deviation; SMC: Specialist medical care; WPAI:GH: Work Productivity and Activity Impairment Questionnaire: General Health V2.0

## Competing interests

Dr Crawley is a medical advisor for the Association for Young people with ME, (AYME) and the Sussex & Kent ME/CFS society. She does not receive any reimbursements, fees, funding or salary from either organisation. The other authors declare that they have no competing interests.

## Authors’ contributions

EC conceived the study, participated in the trial design and coordination and drafted the manuscript. NM, JD and LB designed the qualitative methodology which was conducted by LB. NM supervised the qualitative data analyses. ZD provided ethical advice and reviewed the protocol at each stage. JS designed the statistical methodology. WH designed the economic evaluation. AM contributed to the study design. All authors contributed to and approved the final manuscript.

## Supplementary Material

Additional file 1 Work Productivity and Activity Impairment Questionnaire: General Health V2.0. Click here for file

Additional file 2 Example of Health Resource use questionnaire. Click here for file
