# Supplementary material for: Comparing specialist medical care with specialist medical care plus the Lightning Process® for chronic fatigue syndrome or myalgic encephalomyelitis (CFS/ME): study protocol for a randomised controlled trial (SMILE Trial)
Source: Trials. 2013 Dec 26;14:444. doi: 10.1186/1745-6215-14-444 (PMC3879423; doi:10.1186/1745-6215-14-444)
Supplement: Additional file 1 — Work Productivity and Activity Impairment Questionnaire: General Health V2.0. [file 1745-6215-14-444-S1.pdf]

ID Code: 13digits

CFS/NHS *- Specialist help for ME*

SMILE: WPAI:GH

The following questions ask about the effect of your child's health problems on your ability to work and perform regular activities. By health problems we mean any physical or emotional problem or symptom. *Please fill in the blanks or circle a number, as indicated.*

1. Are you currently employed (working for pay)? ____ NO ____ YES
   If NO, tick "NO" and skip to question 6

   The next questions are about the **past seven days**, not including today.

2. During the past seven days, how many hours did you miss from work because of your child's health problems? *Include hours you missed on sick days, times you went in late, left early, etc., because of your child's health problems. Do not include time you missed to participate in this study.*

   ______HOURS

3. During the past seven days, how many hours did you miss from work because of any other reason, such as vacation, holidays, time off to participate in this study?

   ______HOURS

4. During the past seven days, how many hours did you actually work?

   ______HOURS *(If "0", skip to question 6.)*

ID Code: **13digits**

5. During the past seven days, how much did your child's health problems affect your productivity while you were working?

Think about days you were limited in the amount or kind of work you could do, days you accomplished less than you would like, or days you could not do your work as carefully as usual. If your child's health problems affected your work only a little, choose a low number. Choose a high number if health problems affected your work a great deal.

Consider only how much your child's health problems affected productivity while you were working.

Child's health problems had no effect on my work	0	1	2	3	4	5	6	7	8	9	10	Child's health problems completely prevented me from working

CIRCLE A NUMBER

6. During the past seven days, how much did your child's health problems affect your ability to do your regular daily activities, other than work at a job?

By regular activities, we mean the usual activities you do, such as work around the house, shopping, childcare, exercising, studying, etc. Think about times you were limited in the amount or kind of activities you could do and times you accomplished less than you would like. If your child's health problems affected your activities only a little, choose a low number. Choose a high number if health problems affected your activities a great deal.

Consider only how much your child's health problems affected your ability to do your regular daily activities, other than work at a job
.

Child's health problems had no effect on my daily activities	0	1	2	3	4	5	6	7	8	9	10	Child's health problems completely prevented me from doing my daily activities

CIRCLE A NUMBER
