# Supplementary material for: Comparing specialist medical care with specialist medical care plus the Lightning Process® for chronic fatigue syndrome or myalgic encephalomyelitis (CFS/ME): study protocol for a randomised controlled trial (SMILE Trial)
Source: Trials. 2013 Dec 26;14:444. doi: 10.1186/1745-6215-14-444 (PMC3879423; doi:10.1186/1745-6215-14-444)
Supplement: Additional file 2 — Example of Health Resource use questionnaire. [file 1745-6215-14-444-S2.pdf]

ID Code: **13digits**

CFS/NHS *- Specialist help for ME*

NHS

SMILE Health Resource Use Questionnaire

Please tell us about what health care resources your child has used in the last 6 weeks [for follow up last xxxx weeks since last contacted]. We are interested in your use of NHS services, plus anything you have spent caring for your child, and any other things you have had to spend money on because of your child's illness.

1. Resource Use – Hospitals Visits

1.1 How much contact has your child had at **any hospital** during the last **6 weeks**?

	Number of visits/admissions (if none, write 'zero')
Hospital outpatient clinic	
Accident & Emergency department	
Other hospital appointments – please provide details below:	

If your child has not had any hospital appointments in the last **6 weeks**, please go to question 2**.**

1.2 On average, how much did you and your child spend in travelling to hospital?

For public transport please include the cost of the return fares. For private vehicle, please include parking and estimated fuel costs. If there were no costs, please write zero below.

£____.____ p

ID Code: 13digits

2. Resource Use - Primary & Community Care Visits

2.1 Please could you now tell me about any **primary or community care contacts** your child has had during the last **6 weeks [xx for follow up]**. By primary or community care, we mean any care provided by a GP (family doctor), Community Nurse, NHS Walk-in Centre, NHS Direct telephone consultations, etc. We would like to know **how many** primary & community care consultations, you have had, **where** they took place, and **who** the consultation was with.

	Number of contacts (if none, write 'zero')
**GP SURGERY CONTACTS:**	
General Practitioner at the surgery	
Practice Nurse/Nurse Practitioner at the surgery	
Telephone consultation with doctor at the surgery	
Telephone consultation with nurse at the surgery	
Home visit by the doctor	
**COMMUNITY BASED AND OTHER CONTACTS:**	
School Counsellor	
Walk-in Centre – nurse	
Telephone call to NHS Direct	
Other (e.g. community nurse, health visitor. please specify)	

If your child has not had any primary care or community care appointments in the last **6 weeks**, please go to question 3.

2.2 On average, how much did you and your child spend in travelling to primary or community care?

For public transport please include the cost of the return fares. For private vehicle, please include parking and estimated fuel costs. If there were no costs, please write zero below.

£____.____ p

ID Code: 13digits

3. Medication

3.1 Please tell us about any prescribed medication your child has received in the last **6 weeks**. This may have been prescribed by your GP (family doctor) or by a hospital doctor. If possible, please copy the information from the label on the bottle or packet.

	Name	Date Started	Strength / Dose	Tablet / Injection	Quantity and frequency of doses	Total number of doses in the last 6 weeks
Example	Amitriptyline	01/01/10	10mg	Tablet	One dose once a day	30
Medicine 1						
Medicine 2						
Medicine 3						
Medicine 4						
Medicine 5						

3.2 Please tell us about any **medication you have used** for your child in the last **6 weeks** without a prescription (e.g. over-the-counter at a chemist or supermarket).

	Name	Size of packet / bottle	Proportion used	Cost of packet/bottle
Example	Paracetamol	12 tablets	One third	£____.____ p
Item 1				£____.____ p
Item 2				£____.____ p
Item 3				£____.____ p
Item 4				£____.____ p
Item 5				£____.____ p

ID Code: **13digits**

4. Loss Of Earnings and Expenses

Finally, we'd like to ask you about any **loss of earnings** and **other out-of-pocket expenses** you or your immediate family members have incurred as a result of your child's illness during the past **6 weeks**.

4.1 During the last **6 weeks**, have you or your partner either had to reduce your work and income or been unable to work in order to care for your child? *Yes* 1 ☐ *No* 2 ☐

If yes, approximately how much difference per month has this made on household income? (please tick most appropriate box)

£1 - £100 less per month	1 ☐
£101 - £500 less per month	2 ☐
£501 - £1000 less per month	3 ☐
£1001 - £2000 less per month	4 ☐
£2001 or more per month	5 ☐
Other amount (please state______________)	6 ☐

4.2 Since you child has been ill, have you had to spend more money in order to look after your child for whatever reason? *Yes* 1 ☐ *No* 2 ☐

If yes, on average, how much extra do you spend each month?

£1 - £100	1 ☐
£101 - £500	2 ☐
£501 - £1000	3 ☐
£1001 - £2000	4 ☐
£2001 or more	5 ☐
What did you spend this extra money on? ____________________________	6 ☐

Thank you very much for your time
